# Imaging review of lipomatous musculoskeletal lesions

**DOI:** 10.1051/sicotj/2017015

**Published:** 2017-05-05

**Authors:** Ashley M. Burt, Brady K. Huang

**Affiliations:** 1 Department of Radiology, University of California San Diego 200 West Arbor Drive Mail Code: 8756 San Diego CA 92130 USA

**Keywords:** Lipoma, Soft tissue neoplasm, Bone neoplasm, Neurovascular neoplasm, Synovial neoplasm

## Abstract

Lipomatous lesions are common musculoskeletal lesions that can arise within the soft tissues, bone, neurovascular structures, and synovium. The majority of these lesions are benign, and many of the benign lesions can be diagnosed by radiologic evaluation. However, radiologic differences between benign and malignant lipomatous lesions may be subtle and pathologic correlation is often needed. The use of sonography, computed tomography (CT), and magnetic resonance imaging (MRI) is useful not only in portraying fat within the lesion, but also for evaluating the presence and extent of soft tissue components. Lipomas make up most soft tissue lipomatous lesions, but careful evaluation must be performed to distinguish these lesions from a low-grade liposarcoma. In addition to the imaging appearance, the location of the lesion and the patient demographics can be utilized to help diagnose other soft tissue lipomatous lesions, such as elastofibroma dorsi, angiolipoma, lipoblastoma, and hibernoma. Osseous lipomatous lesions such as a parosteal lipoma and intraosseous lipoma occur less commonly as their soft tissue counterpart, but are also benign. Neurovascular and synovial lipomatous lesions are much rarer lesions but demonstrate more classic radiologic findings, particularly on MRI. A review of the clinical, radiologic, and pathologic characteristics of these lesions is presented.

## Introduction

Musculoskeletal lipomatous lesions form a diverse group of entities which arise from a broad range of tissues and range from benign to malignant. The majority of these lesions arise within the soft tissues, but lipomatous lesions within the bone, neurovascular structures, and synovium are also seen. Many of these lesions demonstrate a classic imaging appearance which can confidently make a diagnosis by imaging alone. However, other lipomatous lesions exist on a spectrum where imaging alone is not enough to differentiate the malignant lesions from the benign. The clinical, imaging, and pathologic characteristics of a variety of these lesions are presented and discussed.

## Soft tissue lipomatous lesions

### Lipoma

Lipomas are benign soft tissue tumors composed of mature adipose tissue. These lesions are the most common soft tissue tumor, making up nearly 50% of all soft tissue tumors [1]. Lipomas are most commonly located within the superficial soft tissues of the extremities, back, and neck. These lesions are usually asymptomatic, but due to the superficial location these lesions commonly present less than 5 cm in size [2]. Lipomas deep to the superficial fascia also occur, but much less frequently. These lesions may be intramuscular or intermuscular and most frequently occur in the lower extremity [1–3]. Additional sites include the trunk, shoulder, and upper extremity. Lipomas within certain locations are rare, i.e. the retroperitoneum, hands, feet, and chest wall, and alternative diagnoses should also be considered for lesions in these locations. Unlike superficial lipomas which do not have a gender predilection, deep lipomas are seen slightly more in males [4]. Deep lipomas are often incidental findings and are larger at presentation than the superficial counterparts due to the lack of aesthetic effect.

**Figure 1.**
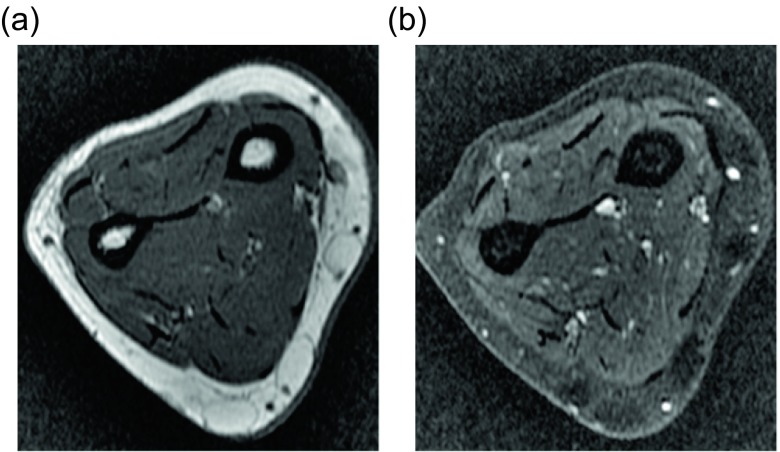
Lipoma: 55-year-old female with multiple palpable masses in the bilateral extremities. Multiple first degree relatives had a history of similar masses. Axial T1W pre-contrast (a) and T1W FS post-gadolinium (b) images reveal multiple, encapsulated masses which follow fat signal on all the sequences.

Though these lesions are usually solitary, they can be multiple in 5–15% of patients [1, 2, 5]. Approximately 30% of these cases are hereditary in nature, a clinically distinct entity called familial multiple lipomatosis (Figure 1); inheritance is most likely polygenic [6]. Patients with the hereditary form of multiple lipomas tend to present at a younger age in the 3rd to 4th decade of life. The lipomas can be very numerous, but the presence of a capsule around the lesion distinguishes them from lipomatosis, which is described as diffuse infiltrative mature adipose tissue.

Superficial lipomas can almost always be diagnosed clinically. In cases where imaging is obtained, lipomas have a characteristic appearance on ultrasound, computed tomography (CT), and magnetic resonance imaging (MRI). Large lipomas may appear as a radiolucency on radiographs, but the finding is not diagnostic. Sonographically, the lesions appear as well-defined, oblong, echogenic mass without posterior acoustic enhancement. In the larger lesions, fine linear striations may be seen coursing parallel to the skin [7]. Deep lipomas are isoechoic or hyperechoic to the adjacent muscle and may have posterior acoustic enhancement due to the greater acoustic transmission within fat compared to muscle. While it has been suggested that ultrasound can establish the diagnosis of a lipoma, studies have shown ultrasound to have a low accuracy [8], and further cross-sectional imaging is often needed.

**Figure 2.**
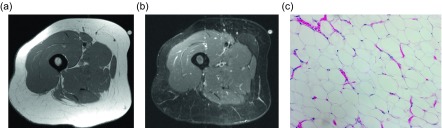
Lipoma: 54-year-old female with proximal right thigh mass. Axial T1W (a) and STIR (b) images reveal an ill-defined mass, intimate with the deep fascia. The mass is isointense to the adjacent fat on T1W images and slightly hyperintense to subcutaneous fat on STIR. Thin internal septa are present without nodularity. H&E 20× (c). Corresponding pathology showed mature adipose tissue with uniform nuclei and no atypia.

**Figure 3.**
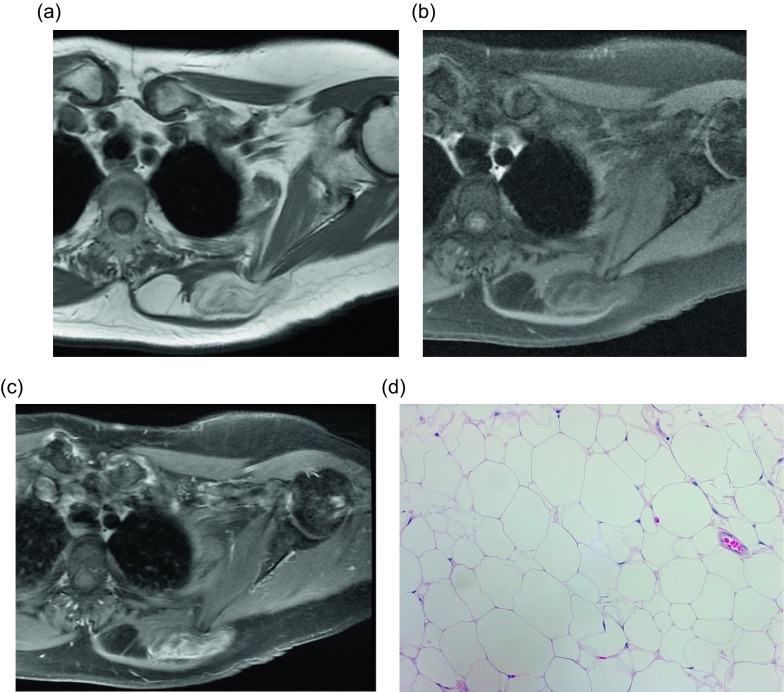
Lipoma: 72-year-old female with history of soft tissue mass posterior to the left scapula. Axial T1W pre-contrast (a), T1W FS (b) and T1W FS post-gadolinium (c) images demonstrate a predominately fat-signal mass in the left posterior chest wall, between the rhomboid and trapezius muscles. Few thin internal septa are seen without nodularity. Heterogeneous enhancement is seen on post contrast images, greater than expected than with a conventional lipoma, which raised concern for an atypical lipomatous tumor or angiolipoma. H&E 20× (d). Corresponding pathology showed mature adipose tissue with uniform nuclei and no atypia, consistent with benign lipoma.

On non-contrast CT, the classic appearance of a lipoma is a circumscribed, homogeneously low (fat) density mass ranging from −120 to −65 Hounsfield units [9, 10]. Similarly, the characteristic lipoma is an encapsulated lesion isointense to the subcutaneous fat on all MRI sequences [11]. Intramuscular lipomas may not be encapsulated and instead may insinuate within the skeletal muscle; this appearance has never been described with liposarcoma. These distinguishing features are reportedly only present in 11–22% of cases [5, 9–11]; this surprisingly low percentage is felt to be related to the fact that most typical lipomas will be clinically diagnosed and will never be evaluated by imaging. Of the imaged lipomas, many demonstrate thin internal septa (Figure 2). These septa usually do not enhance in benign lipomas (58%) compared to marked enhancement in well-differentiated liposarcomas (75%); no non-enhancing septa were seen in malignant lesions [10]. Though the septa are usually less than 2 mm, thick and nodular septa have been reported in up to 31% of lipomas [5, 9–11]; these lesions are indistinguishable from liposarcoma by imaging (Figure 3).

Another complicating feature of some lipomas is mineralization, which is present in up to 11% of imaged lesions [5, 9]. This can appear as either chondroid or osteoid matrix. The lipomas may also undergo necrosis or atrophy. Though lipomas are benign without risk of malignant transformation, if there is atypical appearance by imaging, biopsy should be considered to rule out liposarcoma.

Histopathologically lipomas are encapsulated tumors composed of mature adipocytes. Fibrous connective tissue may be seen which correlates with the thin septa seen on imaging. Lipomas have a rich vascular supply, though the tenuous vessels are often compressed by the large adipocytes. Marginal surgical excision is performed for symptomatic lesions [1, 12]. In a lesion with an atypical appearance or with possible concern for malignancy, the presence of muscle fibers within the lesion can be a helpful indicator of benignity [13]. However, this is not completely diagnostic and if clinical concern is high, further immunohistochemical tests, such as MDM2 and CDK4 expression, should be performed [14]. It is suggested that fluorescence in situ hybridization (FISH) for MDM2 amplification should be considered for recurrent lesions, deep extremity lesions >10 cm, in patients older than 50 years of age, equivocal atypia, retroperitoneal or abdominopelvic lesions, and in certain warranted clinical situations [15].

### Liposarcoma

The main differential consideration for lipomas are liposarcomas, as some of these lesions can have a similar imaging and histologic appearance. Liposarcoma makes up 10–35% of soft tissue sarcomas [12, 16–19], second only to undifferentiated pleomorphic sarcomas (malignant fibrous histiocytoma). Liposarcomas have been further subcategorized by the World Health Organization (WHO) into well-differentiated, myxoid, pleomorphic, mixed, dedifferentiated types.

**Figure 4.**
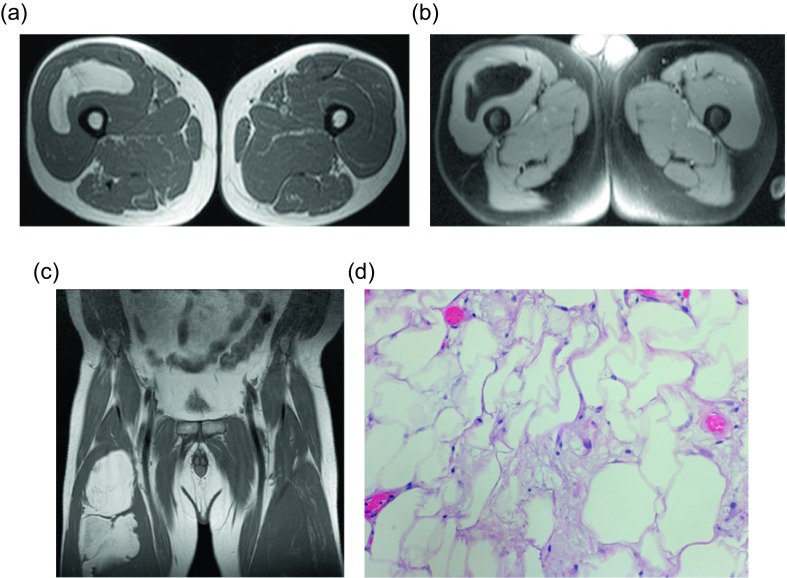
Liposarcoma: 57-year-old man with right thigh mass. Axial (a) and coronal (b) T1W and axial PD FS (c) images show a mass within the vastus intermedius muscle with some thick internal septa. H&E (d). Corresponding pathology revealed numerous variably sized mature adipocytes with focal mild nuclear atypia. Fibrous tissue septa containing spindled cells were also seen.

The most common liposarcoma subtype is a well-differentiated liposarcoma, which makes up approximately half of these lesions [12, 16, 17, 19]. These lesions are also known as atypical lipomatous tumors. Up to 75% of these lesions are found within the deep soft tissues of the extremities [12, 16, 17, 19]. In contradistinction to benign lipomas, deep-seated areas are common sites for liposarcomas, such as the retroperitoneum which accounts for 20–33% of lesions [12, 16, 17, 19]. These often painless, slow-growing masses do not pose a metastatic risk, but may dedifferentiate into a malignant lesion. Histologically these lesions are further separated into five categories, though distinctions between these cannot be identified by imaging. The histologic appearance of the most common subtype, a lipoma-like well-differentiated liposarcoma, demonstrates mostly adipocytes with scattered interspersed lipoblasts. The expression of MDM2 and CDK4 in these lesions can help differentiate this lesion from a classic lipoma [20–23]. By imaging the lesion may appear similar to a lipoma, but care should be taken to identify thick (>2 mm) or irregular septa (Figure 4). The enhancement of these is slightly more pronounced than the thin septa in a lipoma.

**Figure 5.**
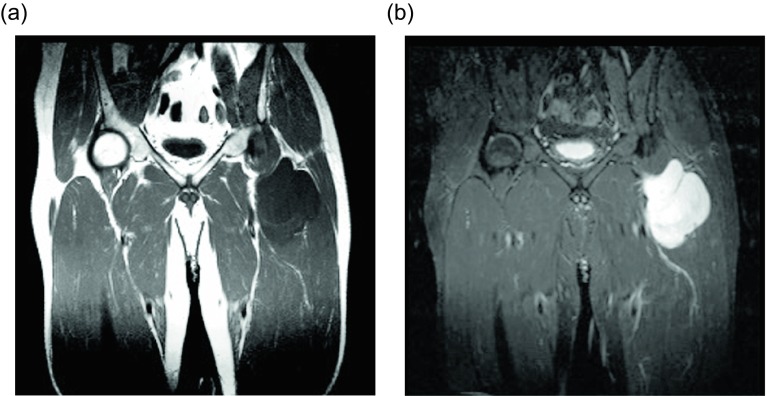
Liposarcoma: 52-year-old male with left thigh mass. Coronal T1W (a) and T2W (b) images show a lobulated mass with low T1 signal and high T2 signal. No definite fat signal is appreciated within the lesion.

Myxoid liposarcoma, the next most common class of liposarcoma, occurs in the intermuscular space in up to 80% of the cases [24]. This subtype encompasses both myxoid tissue and round cell components. Predominately myxoid lesions are gelatinous with a thin plexiform vascular network. The corresponding imaging demonstrates high T2 signal throughout the myxoid portions of the tumor due to the high water content (Figure 5). On T1-weighted imaging, linear or nodular areas of high intensity within the predominately low intensity mass create a marbled appearance; this high T1 signal corresponds with lesional fat. However, mature adipose tissue is scant and may be unidentifiable by imaging or histology in some lesions [21–23, 25]. Occasionally the lesion will appear as a cystic mass with homogeneous high T2 signal. Post-contrast imaging can help distinguish this lesion from a cyst by demonstrating enhancement, though the enhancement pattern and extent varies greatly among lesions. If the lesion is predominately composed of round cells, intermediate signal will be seen on both T1- and T2-weighted imaging.

Less common types of liposarcoma include pleomorphic, dedifferentiated, and mixed-type. Pleomorphic and dedifferentiated subtypes are both high-grade malignancies. Pleomorphic liposarcomas are large, multinodular, well-defined masses. Heterogeneity on imaging is related to internal hemorrhage and necrosis. These lesions are often difficult to diagnose by imaging given their relative lack of adipose tissue [20, 23, 25]. Dedifferentiated liposarcomas share similar imaging features with well-differentiated liposarcoma; however, they also contain soft tissue nodules measuring greater than 1 cm, which represents the dedifferentiated portion of the lesion.

### Elastofibroma dorsi

**Figure 6.**
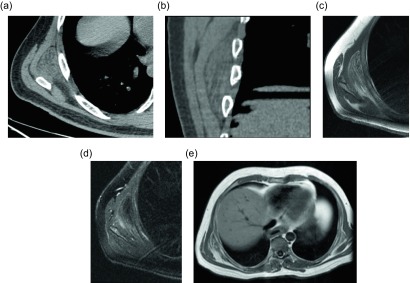
Elastofibroma dorsi: 44-year-old male with a painless mass in the right posteroinferior scapular region. Axial (a) and coronal (b) non-contrast CT demonstrate a poorly-circumscribed mass, deep to the serratus anterior muscle, with tissue of attenuation similar to muscle and internal strands of fat. Subsequent axial T1W (c) and T2W fat saturated (d) images have a similar appearance with signal intensity comparable to muscle and internal bands of fat which suppress on fat saturated images. Larger field of view T1W (e) image from the same study demonstrate a small mass with similar signal intensity within the left subscapular region.

Another benign fat-containing soft tissue lesion is an elastofibroma dorsi. This lesion classically occurs in the infrascapular region, deep to serratus anterior and latissimus dorsi. These lesions are bilateral in up to 60% of cases and when unilateral are more often seen on the right than left [26]. Less commonly, an elastofibroma may be discovered at the ischial tuberosity, olecranon or at another site along the thoracic wall [26]. Though initially thought to be rare, these lesions can be found incidentally in up to 2% of all chest CTs [27]. An autopsy series suggested prevalence approaching 25% in women [28], though these figures may be exaggerated due to the small autopsy sample size. Nevertheless, elastofibromas occur more commonly than initially surmised. CT findings of a poorly defined, soft tissue density lesion with internal fat striations in the infrascapular region are diagnostic for elastofibroma dorsi; the soft tissue component demonstrates similar density to the adjacent skeletal muscle [29]. However, these lesions can appear homogeneous if the internal fat content is low. These lesions may be found on MRI or ultrasound imaging in the work-up of a symptomatic patient. Similar to CT, MRI findings demonstrate a fibrofatty lesion, with the fibrous tissue appearing isointense to skeletal muscle on T1- and T2-weighted imaging (Figure 6). Sonographically these lesions appear as echogenic fibroelastic lesions with internal curvilinear streaks of relatively hypoechoic fat [30, 31]. No treatment is required unless the patient is symptomatic. In those cases, the lesion can be surgically excised; rare reports of recurrence are thought to be related to incomplete excision [29]. On pathology, this non-encapsulated tumor demonstrates eosinophilic collagen bundles, stellate elastic fibers, and a variable amount of mature adipocytes [26].

### Angiolipoma

**Figure 7.**
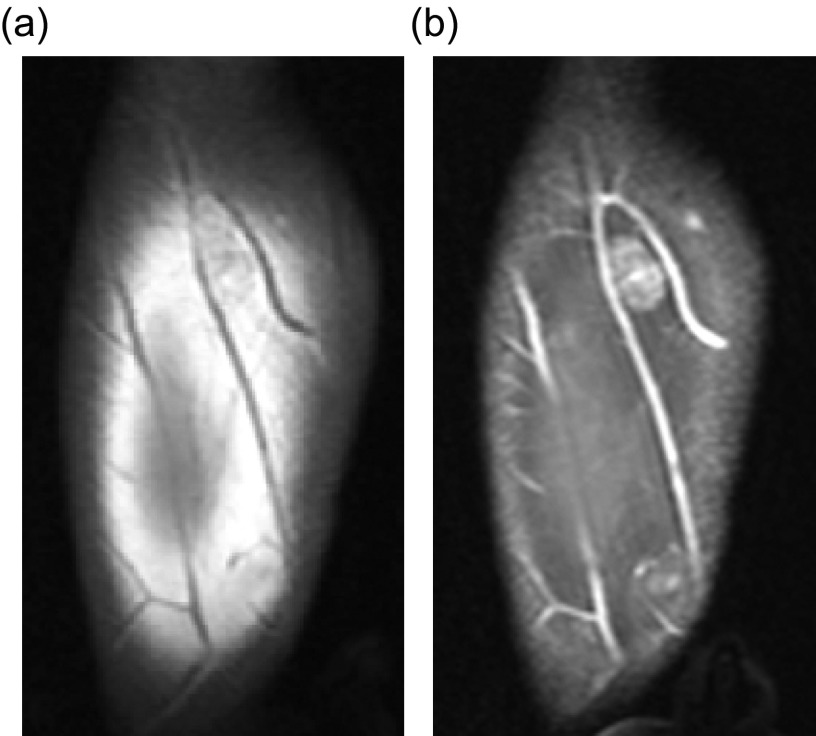
Angiolipoma: 60-year-old male with history of familial lipomatosis status post multiple previous resections. Now with enlarging right arm masses with sharp, radiating pain in the right arm. Coronal T1W pre- (a) and T1W FS post-contrast (b) images depict a heterogeneous subcutaneous mass of the right forearm which predominately isointense to fat with scattered internal low signal intensity foci. Heterogeneous enhancement is present on post-contrast imaging.

One of the many subtypes of a lipoma is an angiolipoma, which is a soft tissue tumor composed of mature fat and small blood vessels. Angiolipomas are also known as hemangiolipomas, vascular lipomas, or fibromyolipomas. Originally, up to 17% of lipomas were felt to be angiolipomas [32], though a subsequent study found angiolipomas to represent only 5% of all lipomas [33]. Typically, these lesions involve the neck, chest wall, and upper extremities. Both non-infiltrating and infiltrating subtypes have been described. The non-infiltrating lesions present as painful, subcutaneous lesions and always demonstrate a thin capsule histologically. These well-circumscribed lesions are typically less than 2 cm and be locally excised without high rate of recurrence [33]. The infiltrating lesions are non-encapsulated or only partially encapsulated and invade the adjacent skeletal muscle and fibrous tissues. The infiltrating lesions may be larger at presentation than the non-infiltrating type and require a wide excision. Imaging appearance will depend on the subtype, but both lesions will contain a mixture of soft tissue and fatty components on all imaging modalities, which may make it difficult to differentiate this lesions from a liposarcoma (Figure 7). Angiographic findings can be misleading because the coarse neovascularity suggests a more aggressive lesion. Rarely, epidural angiolipomas may also occur.

### Lipoblastoma

**Figure 8.**
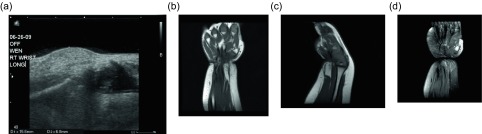
Lipoblastoma: 1-year-old girl with a right wrist mass. Directed ultrasound (a) of the right wrist portrays a well-circumscribed, predominately hyperechoic lesion in the superficial soft tissues. Coronal (b) and sagittal (c) T1W pre-contrast images show a predominately fat-signal lesion centered around the distal flexor carpi radialis tendon with internal low signal intensity components. T1W FS post-gadolinium (d) images demonstrates enhancement of the internal curvilinear components due to the dense capillary network of the myxoid stroma.

**Figure 9.**
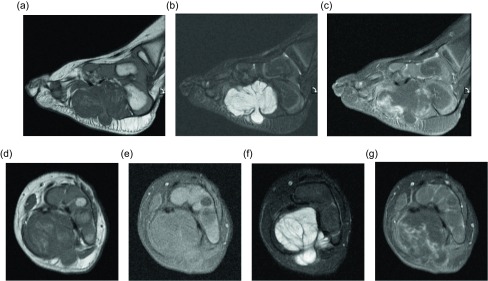
Lipoblastoma: 18-month old boy with left foot mass. Sagittal T1W (a), STIR (b), T1W FS post-contrast (c) images demonstrates a multilobular mass along the medial plantar aspect of the foot. The mass is hypointense on the T1W image and hyperintense on the fluid sensitive images. Portions of the mass demonstrate enhancement on post-contrast imaging. The mass extends through the plantar aponeurosis and lateral plantar fascia. Axial T1W (d), T1W FS (e), T2W FS (f), and T1W FS (g) post-contrast images again show the heterogeneously enhancing mass extending through the plantar aponeurosis. Portions of the mass suppress with fat saturation.

Lipoblastomas are rare, benign mesenchymal tumors, which are discovered almost exclusively before three years of age [34]. Originally, these lesions were reported in the extremities in up to 72% of the cases [34], but more recent studies have described equal incidence of extremity and non-extremity lipoblastomas [35]. Microscopically these lesions demonstrate immature adipose tissue with lipoblasts, capillaries, and myxoid stroma [34], in contradistinction to lipomas which demonstrate mature adipocytes. Imaging appearance depends on the amount of myxoid stroma among the fat; the younger the patient, the more myxoid component present (Figure 8). Enhancement occurs due to the rich capillary network. MRI is the modality of choice in order to define the extent of the mass and the possibility of local invasion (Figure 9). Despite benignity, these lesions may become symptomatic related to the potential for local invasion and ability to grow rapidly in size, though this is uncommon. When local invasion does occur, it is referred to as lipoblastomatosis. These lesions are then surgically excised, with reports of recurrence ranging from 14 to 33% [34, 36], related to incomplete excision. Recent studies have demonstrated a chromosomal rearrangement involving the 8q11-13 region in up to 61% of cases [37], which can be employed to differentiate this lesion from a liposarcoma.

### Hibernoma

**Figure 10.**
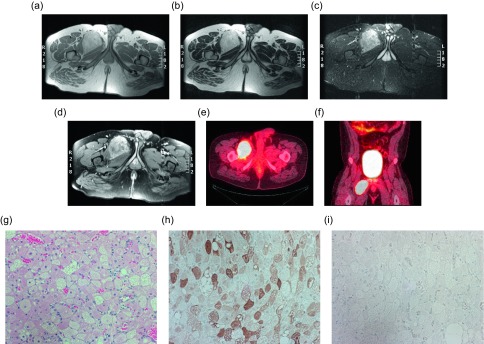
Hibernoma: 29-year-old male with a right thigh mass. Axial T1W (a), T2W (b), STIR (c) and T1W FS post-gadolinium (d) images show a hyperintense mass between the adductor longus and brevis muscles on the right. The signal intensity is similar to the adjacent fat on T1W and T2W; however, on STIR the mass is slightly hyperintense relative to the subcutaneous fat. Post-contrast images demonstrated heterogeneous enhancement. Axial (e) and coronal (f) fused FDG PET-CT demonstrate the marked FDG avidity of the mass. H&E (g), S100 (h), Desmin (i). Tissue obtained demonstrates multlivacuolated, polygonal cells with granular eosinophilic cytoplasm. Immunohistochemical stains are positive for S-100, and negative for desmin.

A hibernoma is a rare lipomatous lesion composed of brown fat affecting middle-aged patients. These lesions most commonly arise in the thigh and subcutaneous tissues of the shoulder, upper back, and neck [38, 39]. Though most of these lesions occur in the subcutaneous tissues, up to 10% have been reported as intramuscular and a few rare cases of intraosseous involvement have been described [40]. Four variants of hibernoma have been described including typical, myxoid, lipoma-like, and spindle cell, in decreasing order of incidence [38]. Like other lipomatous lesions, the imaging appearance of these tumors depends upon the amount of fat within the lesion. The appearance on T1- and T2-weighted imaging is dependent on the lesion subtype. Typical hibernomas demonstrate signal intensity similar, but not identical to the subcutaneous fat, whereas the lipoma-like lesions will more closely match the subcutaneous fat signal intensity. Myxoid-type hibernomas demonstrate more fluid signal intensity. Despite the subtype, these lesions demonstrate prominent low-signal septa on both T1- and T2-weighted images. On post-contrast imaging, diffuse enhancement will be seen that is greater than expected in the other soft tissue lipomatous lesions. Sonographically these lesions will appear nearly identically to lipomas as a hyperechoic, well-defined mass; however, on color Doppler imaging, prominent vascularity helps differentiate the two lesions. On angiography, a hypervascular lesion with an intense blush and foci of arteriovenous shunting may be seen. One of the most distinctive imaging findings to help differentiate the lesion is the avid FDG uptake on positron emission tomography/computed tomography (PET/CT) due to hypervascularity and intense glucose metabolism of the brown fat (Figure 10). Despite these suggestive imaging findings, at times it can be challenging to distinguish a hibernoma from a liposarcoma. Histologically hibernomas appear distinct from a lipoma or liposarcoma. The typical brown fat hibernomas pathologically appear as multivaculoated fat cells arranged in a lobular pattern with a prominent vascular supply. The prominent vascular supply of this lesion accounts for the previously mentioned imaging characteristics that help differentiate a hibernoma from an intermediate-grade or malignant lipomatous tumor. These lesions are not usually symptomatic, but may cause nerve compression or limited range of motion in certain locations. If symptomatic or for aesthetic reasons, these lesions can be removed without risk of recurrence [38, 39].

## Osseous lipomatous lesions

### Parosteal lipoma

**Figure 11.**
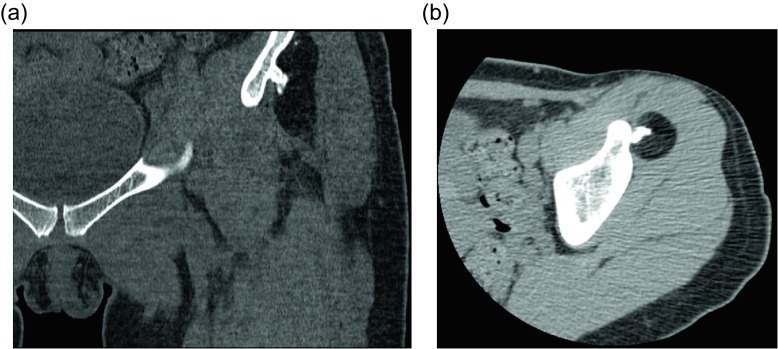
Parosteal lipoma: 45-year-old woman with history of left hip “osteochondroma”. Coronal CT image viewed with bone windows (a) and axial CT image with soft tissue windows (b) show a fat-attenuating lesion within the deep soft tissues of the left thigh. The lesion is intimately associated with the underlying iliac bone and an associated irregular, ossific protuberance arising from the iliac bone.

Parosteal lipomas are benign lesions composed of mature adipose tissue. These lesions are much less common than their soft tissue counterparts, making up only 0.3% of all lipomas [41]. Parosteal lipomas are not merely soft tissue lipomas located adjacent to periosteum, but rather demonstrate intimate association with the periosteum. Osseous reaction is present in the underlying bone in up to 60% of cases [42]. Typically, the reaction is hyperostotic and manifests as cortical thickening, sclerosis, calcification, or formation of an osseous excrescence. If an osseous excrescence is formed, the radiologic appearance may be very similar to that of an osteochondroma; however, the lesion can be differentiated given the lack of medullary continuity (Figure 11). Less commonly the reactive bone change can manifest as smooth cortical scalloping or bowing, but bone destruction has not been reported. The lipoma itself demonstrates imaging findings identical to other soft tissue lipomas on CT and MRI [41]. Occasionally, hyaline cartilage can be seen along the larger osseous protuberances which appear as intermediate T1 and high T2 signal intensity. Fibrous tissue can also be seen, and can be differentiated from cartilage given its low-signal intensity on T2-weighted images. Most commonly these lesions are found along the femur or proximal radius. Motor and sensory deficits may be the presenting symptom due to mass effect on nearby neurovascular structures [42]. Muscle atrophy in a specific nerve distribution can be seen if the lesion is causing nerve compression. Though these lesions share histopathologic features with soft tissue lipomas, parosteal lipomas likely arise from periosteal mesenchymal cells [43]. Some of these lesions are associated with a HMGA2-LPP (high mobility group A2 LIM-containing lipoma-preferred partner) fusion gene secondary to a t(3;12)(q27-28-28;q14-15) translocation [44]. Since this fusion gene is only detectable in benign lesions [44], testing for the gene can be helpful in cases when the diagnosis cannot be made based upon imaging and biopsy alone.

### Intraosseous lipoma

**Figure 12.**
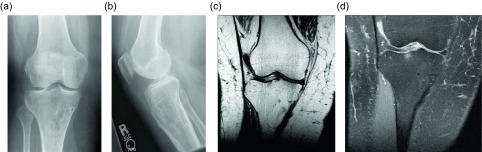
Intraosseous lipoma: 61-year-old female with chronic left knee pain. Frontal (a) and lateral (b) radiographs of the asymptomatic right knee obtained for comparison. Radiographs demonstrate a lucent lesion in the right tibial metaphysis with sclerotic margins and a narrow zone of transition. There is no periosteal reaction or associated soft tissue mass. Coronal T1W (c) and PD FS (d) images show the tibial metadiaphyseal lesion as isointense to fat on T1W with signal loss on the proton density fat saturated image.

**Figure 13.**
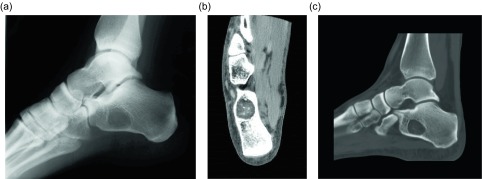
Intraosseous lipoma: 25-year-old female with ankle pain. Lateral radiograph (a) of the right ankle reveal a radiolucent lesion in the anterior portion of the calcaneus with a thin sclerotic border. Axial (b) and sagittal (c) non-contrast CT images again demonstrate the well-circumscribed lesion in the anterior calcaneus with thin sclerotic rim. These images demonstrate the fat attenuation within the periphery of the lesion with central soft tissue density and calcification.

**Figure 14.**
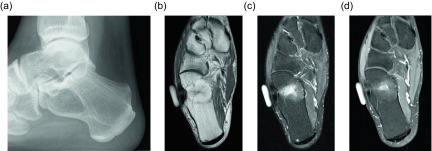
Intraosseous lipoma: 48-year-old male with lucent lesion in the calcaneus on prior radiographs, status post aspiration. Suspected lipoma versus bone cyst. Lateral radiograph (a) of the ankle demonstrates radiolucent lesion at the anterior aspect of the calcaneus. Axial PD (b), PD FS (c) and axial T1 FS post-gadolinium (d) images. The lesion is predominately isointense to fat with loss of signal on fat saturated images. Foci of high signal within the lesion on the PD FS images represents cystic change. On post contrast imaging, mild enhancement of the cystic portions is observed.

**Figure 15.**
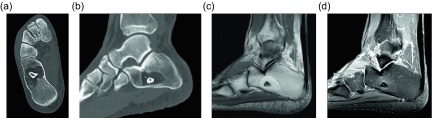
Intraosseous lipoma: 64-year-old male presents for follow-up of an incidentally discovered calcaneal intraosseous lipoma. Axial (a) and sagittal (b) non-contrast CT images demonstrate well-circumscribed lesion in the central calcaneus with thin sclerotic rim and central fat attenuation. Central thick rim of dystrophic calcification produces a classic “bull’s-eye” appearance related to cystic degeneration. Corresponding sagittal T1W (c) and T1W FS post-gadolinium (d) show the predominately fat signal calcaneal lesion with a central region of low T1 signal intensity compatible with cystic change and calcification. Minimal enhancement of the cystic portion of the lesion is present.

Another benign lipomatous lesion is an intraosseous lipoma. Patients are usually within their 4th to 5th decades of life at presentation and classically have focal pain. These lesions occur within the lower extremity up to 71% of the time [45]; the calcaneus is often referred to as a classic location, though there is actually a wide variation of calcaneal prevalence reported within the literature, ranging from 8 to 63% [45]. Radiographically these lesions appear as lucent lesions with partial or complete marginal sclerosis in 74% of patients (Figure 12) [45]. Intralesional calcification is present in more than half the patients and is overwhelmingly dystrophic in appearance and central in location (Figure 13) [45]. Many lesions may mimic an intraosseous lipoma on radiography including fibrous dysplasia, aneurysmal bone cyst, simple cyst, bone infarct, and chondroid tumors (Figure 14). Bone infarcts can be differentiated from intraosseous lipomas by the location of the calcification, which is peripheral and serpentine in bone infarcts and central and dystrophic in intraosseous lipomas. On cross-sectional imaging these lesions demonstrate expected fat density/signal intensity (Figure 15). Histologically these lesions are staged according to the amount of viable adipocytes within the lesion versus necrosis and calcification [46] (Table 1).

**Table 1.** The histologic Milgram staging system of intraosseous lipomas compared to the radiologic findings.

Milgram stage	Histology	Radiology
1	Viable fat cells and ± bony trabeculae	Radiolucent, well defined, expands cortex
2	Viable fat + some fat necrosis + few dystrophic calcifications	Radiolucent, well defined and central calcifications
3	Viable fat + dystrophic calcifications + cyst formation + reactive bone formation	More radiodense with thick sclerotic borders

## Neurovascular lipomatous lesions

### Fibrolipomatous hamartoma

**Figure 16.**
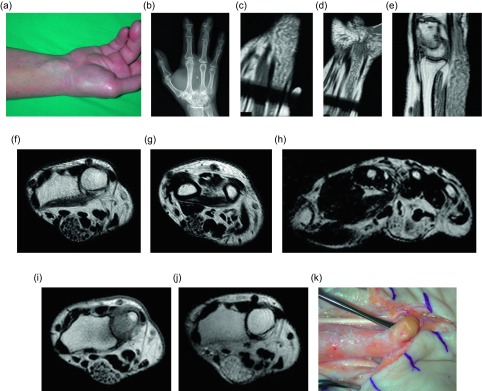
Fibrolipomatous hamartoma: 51-year-old female with prior middle finger resection for congenital macrodactyly, presented with three months of numbness and tingling in the fingers. On physical examination (a), a protruding mass was seen along the course of the median nerve at the carpal tunnel. Corresponding frontal radiograph (b) of the left hand demonstrates postoperative changes from resection of the middle finger with heterotopic bone formation in the surgical bed. Advanced degenerative changes are seen, predominately at the thumb carpometacarpal and distal interphalangeal joints. Coronal (c, d) and sagittal T1W (e) images of the left wrist demonstrate the serpentine appearance of the median nerve fascicles with surrounding fat proliferation. Axial T2W non-fat-saturated images of the left wrist reveal fibrofatty infiltration of the median nerve (f, g). These images demonstrate the “co-axial cable” appearance of the nerve fascicles. The digital nerve branches are also involved (h). Pre- (i) and post-contrast (j) axial T1W images of the left wrist show avid enhancement of the median nerve fascicles following the administration of intravenous gadolinium. Intraoperative image (k) shows a fibrofatty mass of the median nerve at the distal left wrist, corresponding with mass seen on physical examination.

**Figure 17.**
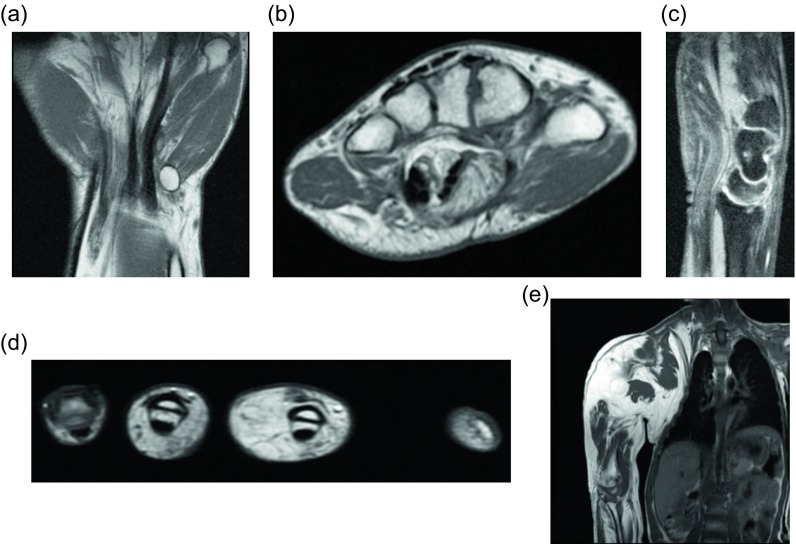
Fibrolipomatous hamartoma: 35-year-old female with congenital macrodactyly of the left hand and history of benign tumor masses requiring multiple surgical excisions. The patient presented with progressive enlargement of the index and ring finger PIP joints with chronic numbness of radial and dorsal left hand and dorsum of index and ring fingers. Coronal (a) and axial (b) T1W non-fat-suppressed images demonstrate fatty proliferation of the median nerve. Postoperative changes from prior middle finger resection are seen on the coronal image. Sagittal T2W fat-saturated (c) image demonstrates diffuse enlargement of the median nerve with bands of low signal intensity, compatible with fatty infiltration of the median nerve. Axial T1W (d) image of the contralateral hand shows lipomatous overgrowth of the middle and ring fingers, in keeping with macrodystrophia lipomatosa. Coronal T1W (e) imaging of this same patient at the age of seven demonstrated marked soft tissue hypertrophy and enlargement of the right upper extremity, consistent with known macrodystrophia lipomatosa.

**Figure 18.**
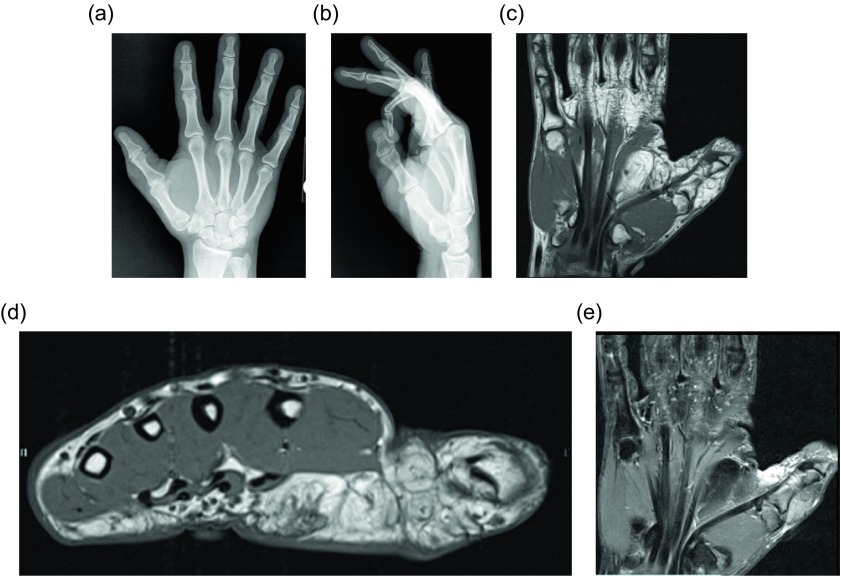
Fibrolipomatous hamartoma: 34-year-old male with slowly enlarging soft tissue mass of the right thumb. Frontal (a) and lateral (b) radiographs of the right hand shows soft tissue fullness of the right first digit and thenar eminence. Coronal (c) and axial (d) T1W and coronal (e) T2W FS (fat-suppressed) images demonstrate thickening of the digital nerve extending to the first digit with lipomatous hypertrophy of the first digit soft tissues.

A fibrolipomatous hamartoma (FLH), also known as lipomatosis of nerve by WHO, refers to adipocyte proliferation within a peripheral nerve. These lesions most commonly occur in infants and children and often present as compressive neuropathy of the affected nerve. The median nerve is involved in up to 50–85% of the cases [47], followed by radial or ulnar nerve involvement. The lower extremity nerves are rarely effected. Fibrolipomatous hamartomas have been associated with macrodystrophia lipomatosa in approximately 25% of patients [48, 49]; macrodystrophia lipomatosa is a disorder of localized gigantism related to mesenchymal overgrowth within specific nerve distribution. Histologically, the adipocyte proliferation occurs around the nerve fascicles, separating the individual nerve bundles (Figure 16). Perineural and endoneural fibrosis can also be seen. This entity is nicely visualized by ultrasound which demonstrates hypoechoic coaxial nerve bundles encased by echogenic infiltrating fat. The MRI findings are pathognomonic; the low-T1 signal intensity nerve fascicles are engulfed by high-T1 signal intensity fat (Figures 17, 18). Relative sparing of the involved nerve may be seen within the carpal and cubital tunnels, likely related to restricted ability for the nerve to expand [48]. Conservative therapy is preferred as long as the patient is asymptomatic.

## Lipomatous lesions within synovial spaces

### Lipoma arborescens

**Figure 19.**
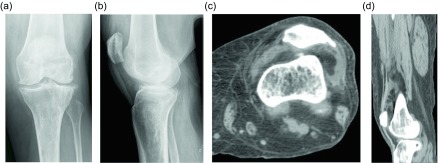
Lipoma arborescens: 59-year-old female with chronic left knee pain. Frontal (a) and lateral (b) radiographs demonstrate tricompartamental osteoarthrosis with fullness of the suprapatellar recess. Axial (c) and sagittal (d) non-contrast CT images reveal a suprapatellar joint effusion with internal small, lobulated masses of fat-attenuation.

**Figure 20.**
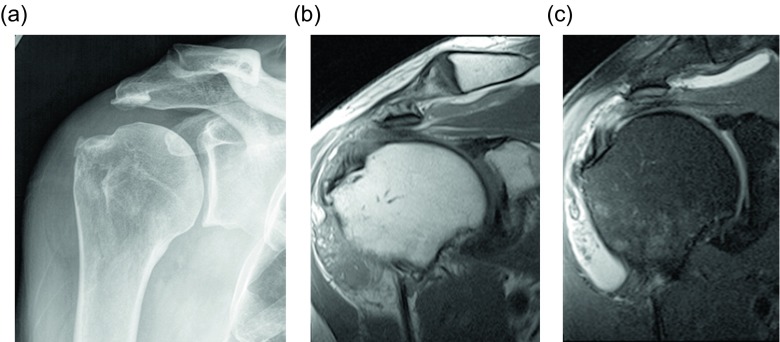
Lipoma arborescens: 70-year-old man with chronic shoulder pain. Frontal radiograph (a) shows a subacromial-subdeltoid effusion. Coronal T1W (b) and T2W FS (c) images demonstrates villous lipomatous proliferation of the synovium outlined by bursal fluid.

**Figure 21.**
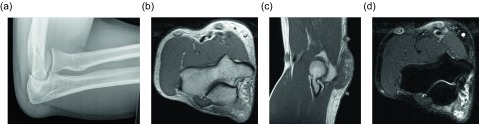
Lipoma arborescens: 42-year-old male with prominent soft tissue over olecranon since injury one year ago. Lateral radiograph (a) of the elbow shows fullness of the olecranon bursa without underlying osseous abnormality. Axial (b) and sagittal (c) T1W and T2W FS (d) images demonstrate frond-like, fatty hypertrophy of the synovium of the olecranon bursa.

Frond-like lipomatous proliferation of the synovium is a benign process known as lipoma arborescens, occurring within joints, bursae, and tendon sheaths. Two categories are reported: the primary form of lipoma arborescens is idiopathic and is much less common than the secondary form, which occurs in the setting of osteoarthritis, rheumatoid arthritis, trauma, or synovitis [4, 50]. Both forms are usually monoarticular, but multifocal or bilateral involvement can occur [51, 52]. The knee is most commonly involved (Figure 19), though the wrist, hip, shoulder, and elbow can also be affected. Rare cases of ankle involvement have been reported [53]. Although these lesions are usually intra-articular, bursal and tendon sheath involvement can occur. Histologically these lesions demonstrate replacement of synovial tissue by mature adipocytes. Radiographic and ultrasound findings are nonspecific, though a joint effusion and synovial hypertrophy may be seen sonographically. Demonstration of infiltrative fat within the synovium is diagnostic, making magnetic resonance imaging the modality of choice. The signal intensity of the lesion should mirror that of fat on all sequences. Joint effusions and bone erosions may be seen [50]. The synovium may enhance on post-contrast imaging due to chronic inflammation. The frond-like appearance of the hypertrophied synovium is the basis of the name lipoma arborescens (Figures 20, 21). Treatment for this benign lesion is synovectomy. However, if the underlying inflammatory etiology persists, the lesion may recur, though reported cases of recurrence are uncommon (Table 2).

**Table 2.** Summary of the sonographic, CT and MRI findings of musculoskeletal fat-containing lesions.

Lesion	Ultrasound	CT	MRI
Lipoma	Well defined Oblong or round Echogenic No posterior acoustic enhancement	Circumscribed Hypodense (−120 to −65 HU) Non-enhancing, thin septa (<2 mm)	Circumscribed Fat signal intensity on all sequences Non-enhancing, thin septa (<2 mm)
Well-differentiated liposarcoma	Similar appearance to lipoma Deep soft tissues/retroperitoneum	Similar to lipoma except septa may be irregular or thicker (>2 mm)	Similar to lipoma except septa may be irregular or thicker (>2 mm), with enhancement
Dedifferentiated liposarcoma		Similar to well-differentiated liposarcoma, plus a soft tissue nodule >1 cm	Similar to well-differentiated liposarcoma, plus a soft tissue nodule >1 cm
Myxoid liposarcoma			Low T1 signal with internal linear/nodular high T1 signal High T2 signal in myxoid portions Occasionally, entirely cystic (high T2)
Pleomorphic liposarcoma	Large Well-defined Heterogeneous	Heterogeneous related to internal hemorrhage and necrosis	Heterogeneous related to internal hemorrhage and necrosis
Elastofibroma dorsi	Infrascapular Bilateral (60%) Echogenic Internal curvilinear hypoechoic streaks	Infrascapular Bilateral (60%) Poorly defined Soft tissue density (isodense to muscle) with internal fat striations	Infrascapular Bilateral (60%) Poorly defined Soft tissue intensity (isointense to muscle) with internal fat striations
Angiolipoma	Circumscribed or infiltrative Small (<2 cm) Similar to liposarcoma May have mild internal vascularity	Circumscribed or infiltrative Small (<2 cm) Heterogeneous soft tissue and fat density lesion	Circumscribed or infiltrative Small (<2 cm) Heterogeneous lesion with soft tissue and fat signal intensity
Lipoblastoma/lipoblastomatosis		Circumscribed or infiltrative May appear similar to liposarcoma Avidly enhancing	Circumscribed or infiltrative Variable appearance based upon myxoid component Myxoid components demonstrate high T2 signal Avidly enhancing
Hibernoma	Similar to lipoma except prominent vascularity on color Doppler imaging	Variable appearance depending on fat contentAvid FDG uptake on PET/CT	Variable appearance depending on fat content Low T1 and T2 signal septa Avid enhancement
Parosteal lipoma	Radiographically: Radiopaque soft tissue mass Adjacent cortical thickening, sclerosis, periosteal reaction, osseous excrescence formation or smooth cortical scalloping	Identical to soft tissue lipoma Adjacent cortical thickening, sclerosis, periosteal reaction, osseous excrescence formation or smooth cortical scalloping	Identical to soft tissue lipoma Adjacent cortical thickening, sclerosis, periosteal reaction, osseous excrescence formation or smooth cortical scalloping Hyaline cartilage (intermediate T1/high T2 signal intensity) may be seen along the osseous excrescence Fibrous tissue (intermediate T1/low T2 signal intensity) may also be present
Intraosseous lipoma	Radiographically: Lucent intramedullary lesion Partial or complete sclerotic rim Dystrophic calcification (>50%)	Fat density intraosseous lesion Partial or complete sclerotic rim May be expansile	Isointense to subcutaneous fat Signal loss on fat suppression sequences Signal void in calcified regions If necrosis, low T1/high T2 signal If cystic, low T1/high T2 signal with peripheral enhancement
Fibrolipomatous hamartoma	Hypoechoic coaxial nerve bundles encased by echogenic fat	Increased fat density along a nerve Mass effect on nearby structures	Low T1 signal nerve fascicles engulfed by high T1 fat intensity Nerve within regions with restricted expansion (e.g. carpal and cubital tunnels) may be spared Nerve bundles, but not surrounding fat, will enhance
Lipoma arborescens		Fat density synovial mass	Frond-like appearance High T1 signal matches subcutaneous fat Signal loss with fat suppression Synovium will enhance, but fat will not

## Conclusion

Lipomatous lesions are common throughout the musculoskeletal system and encompass both benign and malignant lesions. Though the radiographic manifestation of the lesion may be helpful in the diagnosis, radiographic findings can overlap among various lesions and histologic diagnosis may be necessary. Imaging can help in management by targeting the biopsy to the non-lipomatous components of the mass. Ultimately, the understanding of the wide range of musculoskeletal lipomatous lesions and their radiologic appearance is essential to the appropriate treatment of patients.

## Conflict of interest

The authors declare no conflict of interest in relation with this paper.
